# Effects of Intraoperative Ozone Application on Early Implant Stability: A Randomized Split-Mouth Clinical Trial

**DOI:** 10.3390/jfb17080414

**Published:** 2026-08-18

**Authors:** Zeliha Başak Çakır Erdil, Hüseyin Akıllı, Şahin Altuğ, Metin Çalışır

**Affiliations:** Department of Periodontology, Faculty of Dentistry, Adıyaman University, Adıyaman 02040, Türkiye693hsyn@gmail.com (H.A.);

**Keywords:** dental implants, resonance frequency analysis, osseointegration, ozone

## Abstract

Background/Objectives: The aim of this study was to evaluate the effect of intraoperative gaseous ozone applied to the implant osteotomy immediately before implant placement on early implant stability using a split-mouth design and resonance frequency analysis (RFA). Methods: This prospective, randomized controlled split-mouth study included 40 patients receiving 106 implants in bilaterally symmetrical edentulous sites. Patients and outcome assessors were blinded. In the ozone group, ozone gas was applied to the implant site for 60 s immediately before implant placement; the control group underwent the same surgical protocol without ozone application. The primary outcome was the change in implant stability quotient from baseline to three months (ΔISQ). Secondary outcomes included baseline and three-month ISQ values, insertion torque, and postoperative pain assessed using a visual analog scale. Results: All 106 implants were analyzed. No significant between-group differences were observed in baseline ISQ (β = −0.49; 95% CI: −2.73 to 1.75; *p* = 0.668) or insertion torque (β = −0.62 Ncm; 95% CI: −1.96 to 0.72; *p* = 0.362). At three months, ISQ was higher in the ozone group (β = 1.91; 95% CI: 0.75 to 3.06; *p* = 0.001). ΔISQ was also significantly greater in the ozone group (β = 2.40; 95% CI: 0.34 to 4.45; *p* = 0.022; Cohen’s d = 0.42). Implant stability increased significantly in both groups (both *p* < 0.001). Postoperative pain did not differ between groups (β = −0.23; 95% CI: −0.83 to 0.36; *p* = 0.446). Although the effect direction was consistent across sensitivity analyses, ΔISQ was not statistically significant in the patient-level paired analysis (*p* = 0.089). No adverse events were reported. Conclusions: Intraoperative ozone application was associated with a modest increase in early implant stability without a significant difference in postoperative pain. However, given the small effect size and sensitivity of the findings to the analytical method, further large-scale, multicenter randomized controlled trials are required. Clinically, intraoperative ozone may be considered a potential adjunct to standard implant placement protocols, but the current evidence is insufficient to support its routine use.

## 1. Introduction

The rehabilitation of missing teeth with implant-supported prostheses has become an established treatment option in contemporary dentistry owing to its predictable long-term outcomes. Treatment success depends on osseointegration, defined as the establishment of a direct structural and functional connection between the implant surface and the surrounding bone, particularly during early osseointegration, when primary and secondary implant stability interact in a complementary manner [1]. Primary stability represents the mechanical anchorage achieved during implant placement, whereas secondary stability develops through new bone formation during healing. During the first weeks, primary stability decreases before secondary stability has fully developed, creating a critical period in which overall stability temporarily declines. Because early implant failures frequently occur during this period, early stability is influenced not only by initial mechanical anchorage but also by bone quality, implant geometry, micromobility, and bone healing [1,2].

Objective monitoring of the transition from mechanical to biological anchorage during early osseointegration is clinically important. Resonance frequency analysis (RFA) and the resulting implant stability quotient (ISQ) are widely used to monitor early osseointegration, providing a noninvasive, objective, and reproducible assessment of implant–bone complex stability [3,4,5]. The high measurement repeatability and interobserver agreement of the Osstell system support its reliability for longitudinal assessment of implant stability [6].

The quality of osseointegration is also influenced by the implant surface and the microenvironment surrounding the osteotomy. Because early microbial contamination of the surgical site may adversely affect peri-implant healing, adjunctive approaches supporting surface and site decontamination may promote healing by reducing the biofilm burden. However, current evidence does not demonstrate the clinical superiority of any mechanical, chemical, or physical decontamination method, and no universally accepted gold standard has been established [7,8]. This uncertainty has increased interest in biocompatible alternative approaches that do not promote antimicrobial resistance.

Ozone (O_3_) is a potent oxidizing agent composed of three oxygen atoms and exhibits broad-spectrum antimicrobial activity at low medical concentrations without contributing to antimicrobial resistance [4,9]. Experimental models have demonstrated that ozonated water and ozone gas effectively reduce the viability and disrupt the cell membrane integrity of oral microorganisms, including periodontal pathogens, particularly *Porphyromonas gingivalis* [9,10]. In addition to its antimicrobial activity, ozone has been reported to activate antioxidant and anti-inflammatory pathways through controlled oxidative stimulation at low doses. It may also enhance osteoblast proliferation and the expression of osteocalcin and bone morphogenetic proteins, reduce RANKL levels, increase OPG levels, and promote VEGF-mediated angiogenesis [11,12,13]. Collectively, these biological effects suggest that ozone may support bone healing and thereby contribute to the development of secondary implant stability. Moreover, its greater biocompatibility with gingival fibroblasts compared with chlorhexidine, a commonly used antiseptic, makes ozone a biologically promising option for application at surgical sites [14].

Clinical evidence evaluating the effects of ozone application on early implant stability and osseointegration remains limited and heterogeneous. Most previous studies have investigated ozone for surface decontamination in the treatment of peri-implantitis, whereas controlled clinical trials assessing the early healing period using objective stability measurements remain scarce [8]. Furthermore, insufficient standardization of patient- and surgery-related variables in these studies makes it difficult to determine whether the observed effects are attributable to ozone application or interindividual variability [9]. To our knowledge, no clinical study has evaluated the effect of ozone applied directly to the implant osteotomy during surgery on early implant stability using objective RFA measurements and a split-mouth design with the contralateral side serving as an intra-patient control.

This study aimed to objectively evaluate the effect of intraoperative ozone disinfection applied to the implant osteotomy immediately before implant placement on early implant stability using a within-patient split-mouth design and resonance frequency analysis (RFA). To minimize the effects of individual biological and surgical variability, patient selection and the surgical protocol were standardized, and the change in implant stability quotient from baseline to three months (ΔISQ) was defined as the prespecified primary outcome. The null hypothesis of this study was that intraoperative gaseous ozone application before implant placement would have no significant effect on early implant stability compared with the control treatment.

## 2. Materials and Methods

### Study Design and Participants

This study was conducted in accordance with the Declaration of Helsinki and was approved by the Clinical Research Ethics Committee of Harran University (approval no. HRÜ-25.18.55). The study was registered at ClinicalTrials.gov (Identifier: NCT07687680).

Only patients requiring implant-supported rehabilitation in at least two bilaterally symmetrical edentulous sites were included. The split-mouth design allowed each participant to serve as their own control, with ozone application assigned to an implant site on one side and the conventional protocol to the contralateral implant site.

Treatment allocation was determined preoperatively using simple randomization, and the allocation sequence was concealed in sequentially numbered, opaque, sealed envelopes. Immediately before implant placement, M.Ç. opened the corresponding envelope and informed the surgeon of the treatment protocol assigned to each implant site. Consequently, the surgeon (Z.B.Ç.E.) was not blinded to treatment allocation. To ensure standardization of the surgical protocol, all implant placement procedures were performed by the same surgeon. Patients remained unaware of treatment allocation. Implant stability measurements and other clinical assessments were performed by two calibrated independent investigators (H.A. and Ş.A.) who were blinded to group allocation and were not involved in the surgical procedures.

**Figure 1:**
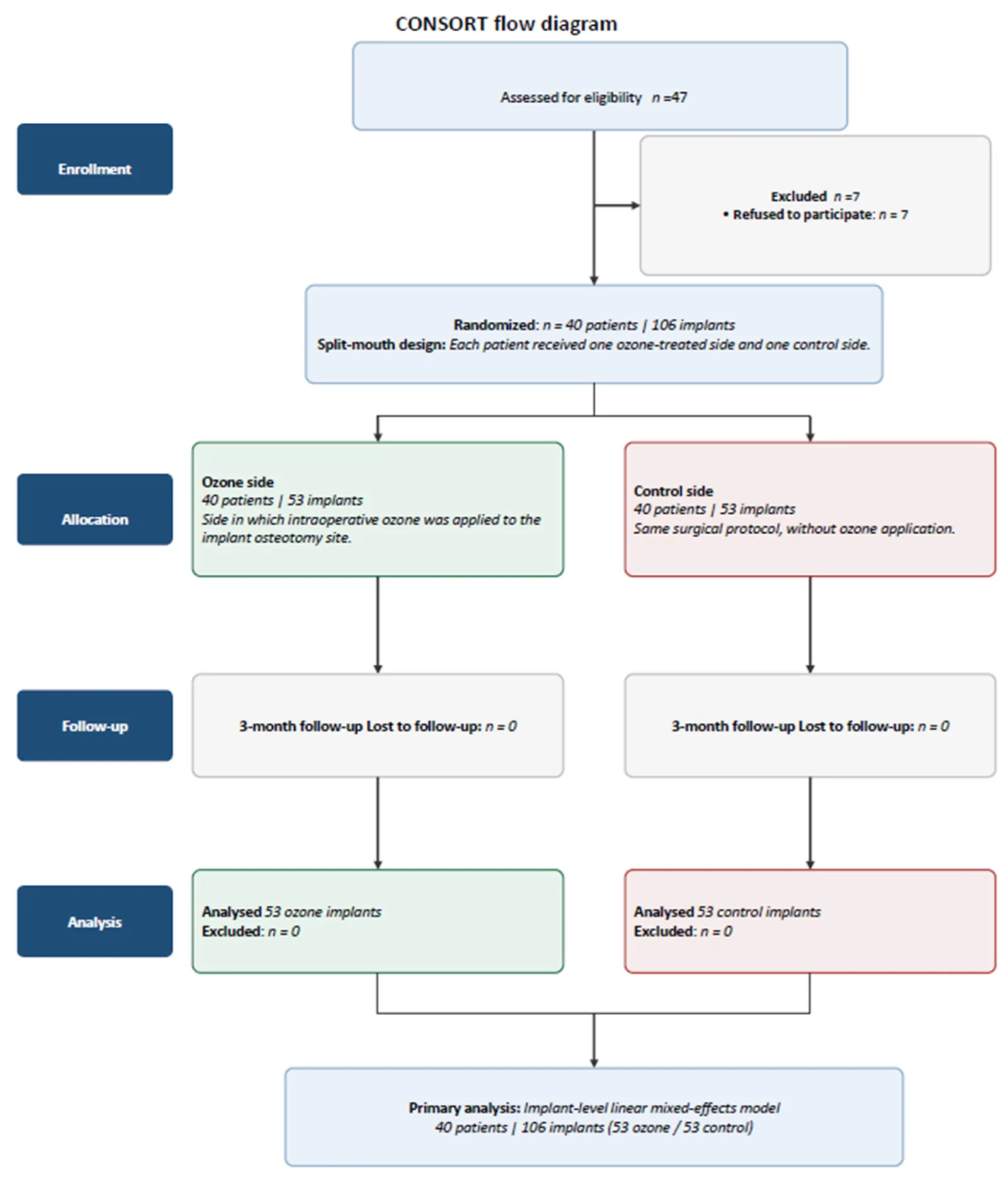
CONSORT flow diagram of participant enrollment, allocation, follow-up, and analysis.

This randomized controlled trial was reported in accordance with the CONSORT reporting guidelines. The participant flow is presented in the CONSORT flow diagram (Figure 1).

**Inclusion Criteria**

Individuals aged 18–65 years were assessed for eligibility. Medically healthy patients requiring implant-supported rehabilitation in at least two comparable bilaterally positioned edentulous sites within the same jaw were included. Eligible participants were required to have sufficient native bone volume to permit implant placement without simultaneous bone augmentation and a keratinized tissue width of >2 mm. Only patients who provided written informed consent and were able to attend all scheduled follow-up visits were enrolled. Candidates with any local or systemic condition considered likely to adversely affect implant healing or prevent completion of the study protocol were excluded.

**Exclusion Criteria**

Patients were excluded if they had an American Society of Anesthesiologists (ASA) physical status classification of III or higher, uncontrolled diabetes mellitus, uncontrolled periodontal disease, a history of bruxism, a diagnosis of osteoporosis, current use of bisphosphonates or other antiresorptive medications, ongoing immunosuppressive therapy, pregnancy or lactation, inadequate oral hygiene, a history of radiotherapy to the head and neck region, or current tobacco use. Patients requiring simultaneous hard- or soft-tissue augmentation at the time of implant placement were also excluded.

**Implant Placement and Ozone Application**

All implants were placed in posterior sites (tooth regions 4, 5, 6, and 7). Within each patient, treatment sites were matched within the same jaw; maxillary sites were paired only with contralateral maxillary sites and mandibular sites only with contralateral mandibular sites. To maintain regional comparability, premolar sites were matched with contralateral premolar sites and molar sites with contralateral molar sites. Preoperative CBCT examinations were obtained for all patients and used for site-specific implant planning. Implant sites were selected and planned to ensure approximately 1.5 mm of buccal bone thickness around the implant.

All surgical procedures were performed by the same surgeon (Z.B.Ç.E.) under local anesthesia with 2% lidocaine hydrochloride containing 1:80,000 epinephrine. Before surgery, all participants rinsed with 0.12% chlorhexidine gluconate for 30 s. Following a midcrestal incision, a full-thickness mucoperiosteal flap was elevated, and the implant osteotomies were prepared under copious sterile saline irrigation according to the manufacturer’s drilling protocol. Internally connected, sandblasted and acid-etched implants (bredent medical GmbH & Co. KG, Senden, Germany) were used in all patients. To standardize implant-related variables, all implants had a diameter of at least 4.0 mm and a length of at least 10 mm. All implants were placed 0.5 mm subcrestally, and the same drilling, placement depth, and insertion protocol were applied to both the ozone and control sides.

According to the split-mouth randomization, ozone was applied to one implant site, while the contralateral site served as the control. In the ozone group, topical ozone was applied to the prepared implant osteotomy immediately before implant placement using a dental ozone generator (OzoneDTA^®^, APOZA Enterprise Co., Ltd., New Taipei City, Taiwan). Ozone was delivered for 60 s at power level 15 through a No. 4 conical probe. The probe was maintained approximately 1 mm from the osteotomy walls without contacting the surrounding tissues, and high-volume suction was used throughout the procedure in accordance with the manufacturer’s recommendations. In the control group, the same surgical protocol was followed without ozone application.

Following implant placement, cover screws were placed on all implants, and primary wound closure was achieved using 4-0 polyglactin 910 sutures. Postoperative care was standardized for all participants. Paracetamol (500 mg; maximum daily dose, 2 g) was prescribed as needed for pain control, and participants were instructed to use a 0.12% chlorhexidine gluconate oral spray twice daily for 14 days. Sutures were removed on postoperative day 14.

**Assessment of Implant Stability**

**Figure 2:**
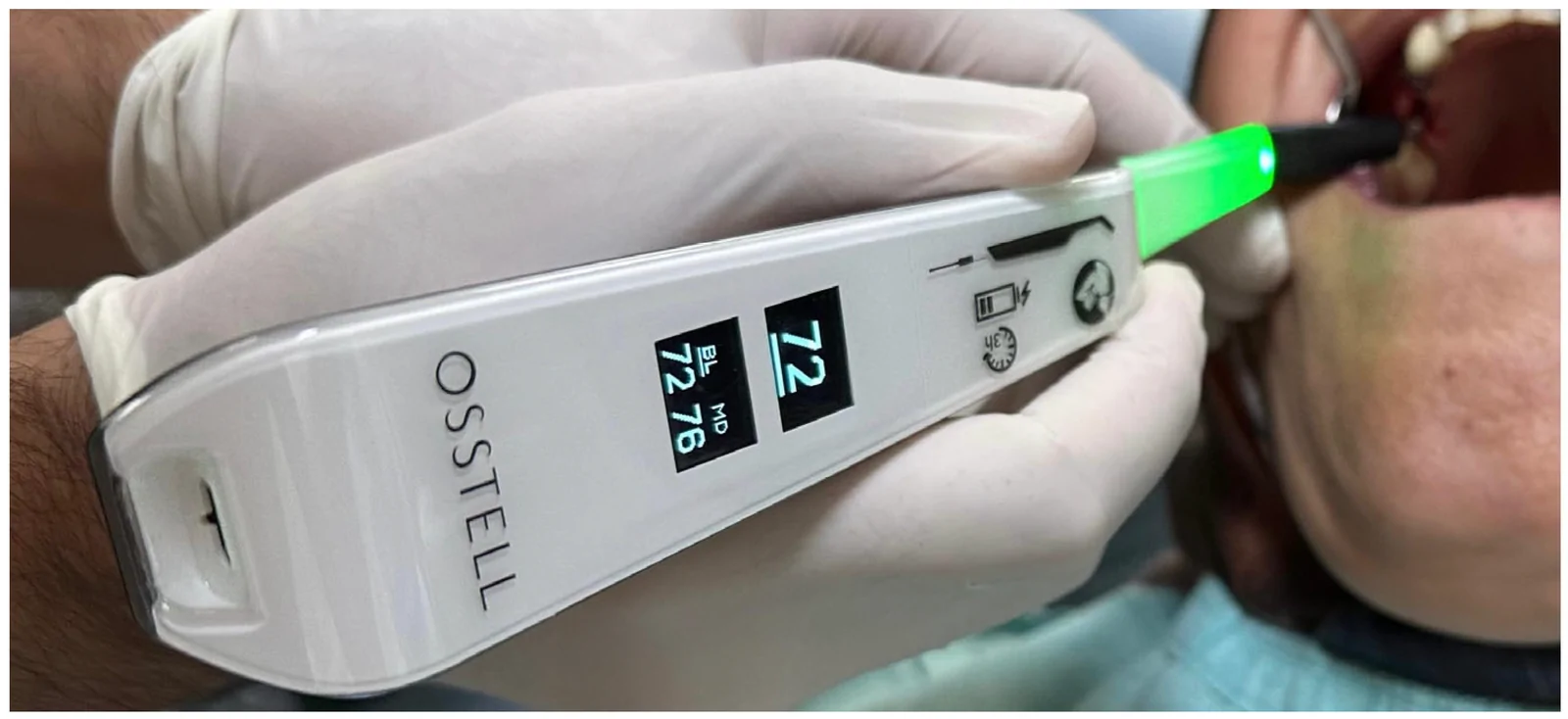
Measurement of implant stability using resonance frequency analysis (RFA).

Implant stability was assessed by resonance frequency analysis (RFA) using the Osstell Beacon device (Osstell AB, Gothenburg, Sweden) and the corresponding SmartPeg transducers in accordance with the manufacturer’s recommendations (Figure 2). Baseline implant stability measurements were obtained immediately after implant placement and before placement of the cover screw.

For each implant, implant stability quotient (ISQ) values were measured in the buccolingual and mesiodistal directions by two independent calibrated investigators (H.A. and Ş.A.) using the same standardized measurement protocol. Each investigator performed one measurement in each direction. The mean of the four measurements obtained (two investigators × two directions) was used as the final ISQ value in the statistical analyses.

Implant stability was assessed at two time points: immediately after implant placement (baseline) and at three months postoperatively. The mean ISQ values obtained at both time points were used in the statistical analyses. Insertion torque (Ncm) was recorded at implant placement using a manual torque wrench. Postoperative pain was self-reported 24 h after surgery using a 0–10 visual analog scale (VAS).

**Statistical Analysis**

Under the split-mouth design, each patient was assigned an ozone-treated side and a control side. Because some patients received more than one implant per treatment side, the resulting data had a clustered structure, with multiple implant-level observations nested within patients. The dataset comprised 106 implants placed in 40 patients. This hierarchical structure was explicitly considered in the statistical analyses, which included both implant-level models accounting for within-patient clustering and patient-level paired comparisons. Continuous variables are presented as mean ± standard deviation (SD).

The sample size was calculated a priori based on the prespecified primary outcome (ΔISQ), with the patient considered the independent unit for the paired split-mouth design. Assuming a two-sided significance level of 0.05 (α = 0.05), 80% statistical power (1 − β = 0.80), and a standardized paired effect size of Cohen’s dz = 0.45, a minimum of 40 participants was required. The sample size calculation was performed using G*Power software (version 3.1).

The primary outcome was the change in implant stability quotient from baseline to three months (ΔISQ). Secondary outcomes were ISQ values at baseline and three months, insertion torque, and postoperative pain assessed using a visual analog scale (VAS).

At each assessment, ISQ was measured in the mesiodistal and buccolingual directions by two independent calibrated investigators. The mean of the four measurements obtained for each implant was used in the analyses.

Between-group differences were estimated using linear mixed-effects models that included treatment group as a fixed effect, with the control group as the reference, and a patient-specific random intercept. Because the data structure did not permit reliable estimation of a patient-level random treatment slope, no random-slope term was included.

The primary analysis was conducted at the implant level using a linear mixed-effects model with a patient-specific random intercept to account for the correlation of multiple implants contributed by the same patient. Given the paired nature of the split-mouth design and the presence of multiple implants per treatment side in some patients, a patient-level paired analysis was additionally performed by averaging implant-level outcomes within each treatment side for each patient. An ordinary least-squares regression model with standard errors clustered at the patient level was also performed as a complementary analysis.

Models were estimated using restricted maximum likelihood (REML). Fixed-effect estimates (β = ozone − control) are presented with 95% confidence intervals and were evaluated using Wald z-tests. Within-group changes from baseline to three months were analyzed using linear mixed-effects models with a patient-specific random intercept.

Model assumptions were assessed for all linear mixed-effects models. Residual normality was evaluated using the Shapiro–Wilk test, and the normality of the random effects was examined using empirical Bayes estimates. Homogeneity of residual variances between treatment groups was assessed using Levene’s test. Residual-versus-fitted-value plots were also visually inspected for potential deviations from model assumptions.

For secondary outcomes showing deviations from model assumptions, inferences were based primarily on patient-clustered robust standard errors (sandwich estimator; CR1). The robustness of the mixed-effects model results was additionally assessed using two alternative approaches: (i) ordinary least-squares (OLS) models with standard errors clustered at the patient level and (ii) paired patient-level analyses based on the mean for each treatment side within each patient, using either a paired *t*-test or the Wilcoxon signed-rank test according to the Shapiro–Wilk test results.

Standardized between-group effect sizes were calculated using Cohen’s d. Baseline comparisons were performed to assess comparability between the treatment groups. Secondary outcomes were analyzed without adjustment for multiplicity. Statistical significance was set at *p* < 0.05. Analyses were performed in Python 3 using the NumPy, SciPy, and statsmodels libraries.

## 3. Results

A total of 40 patients receiving 106 implants were included. The mean age was 46.0 ± 11.1 years (range, 24–65 years), with equal numbers of women and men. In the split-mouth design, the same 40 patients contributed 53 implants to each treatment side. The number of implants contributed per patient was balanced between treatment sides; on each side, 32 patients contributed one implant, five contributed two implants, one contributed three implants, and two contributed four implants. Jaw distribution was identical between treatment sides (32 maxillary and 21 mandibular implants per side). Regarding implant dimensions, 101 of the 106 implants had a diameter of 4.0 mm, whereas the remaining five implants had a diameter of 4.5 mm. In terms of implant length, 99 implants were 10 mm long and the remaining seven were 12 mm long. Treatment-specific implant characteristics are presented in Table 1.

**Figure 3:**
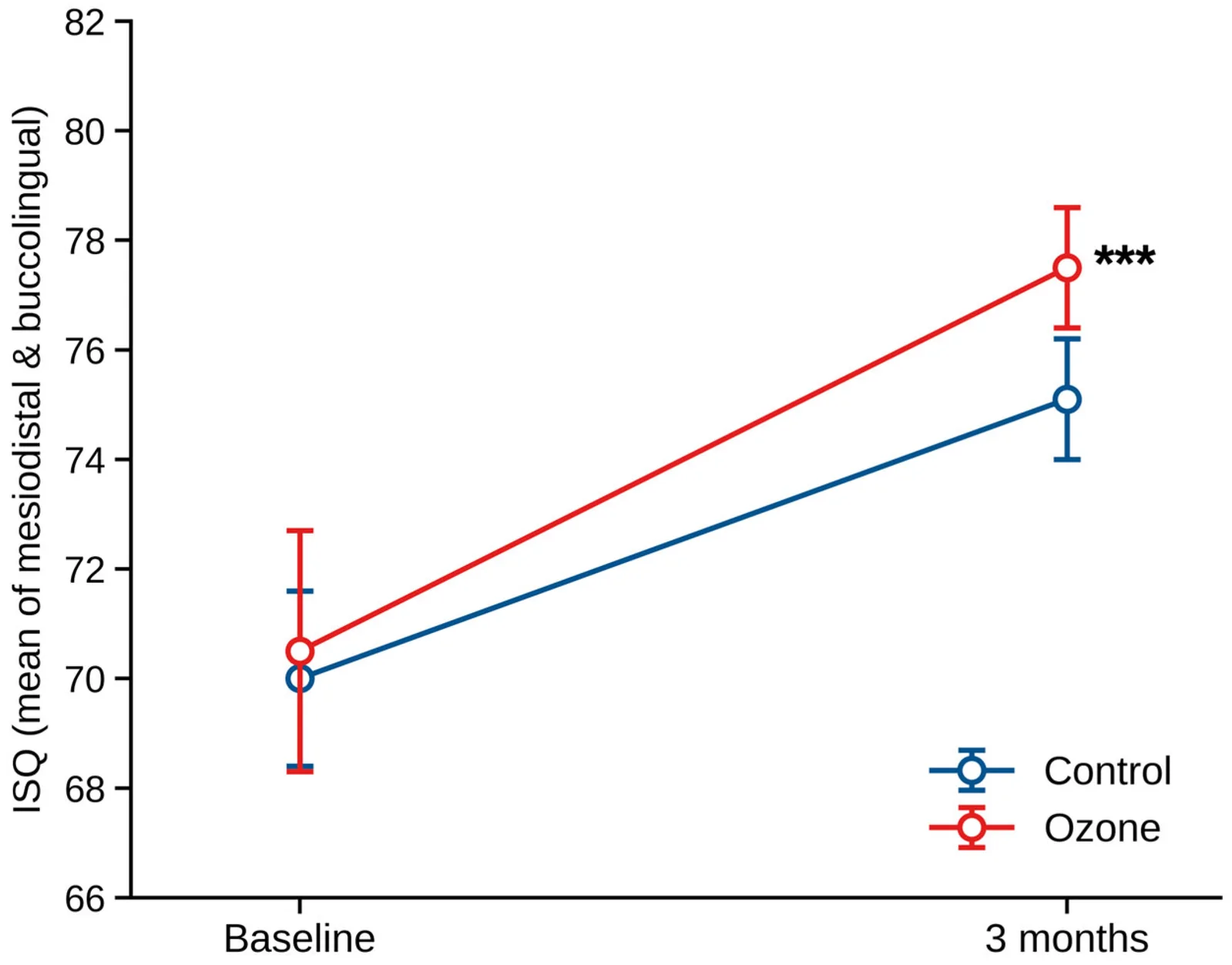
Mean ISQ at baseline and 3 months by group (patient-level means; error bars = 95% CI). Both groups showed significant increases in ISQ from baseline to 3 months (both *p* < 0.001). At 3 months, mean ISQ was significantly higher in the ozone group than in the control group. *** indicates the between-group difference at 3 months (linear mixed-effects model, *p* = 0.001).

**Figure 4:**
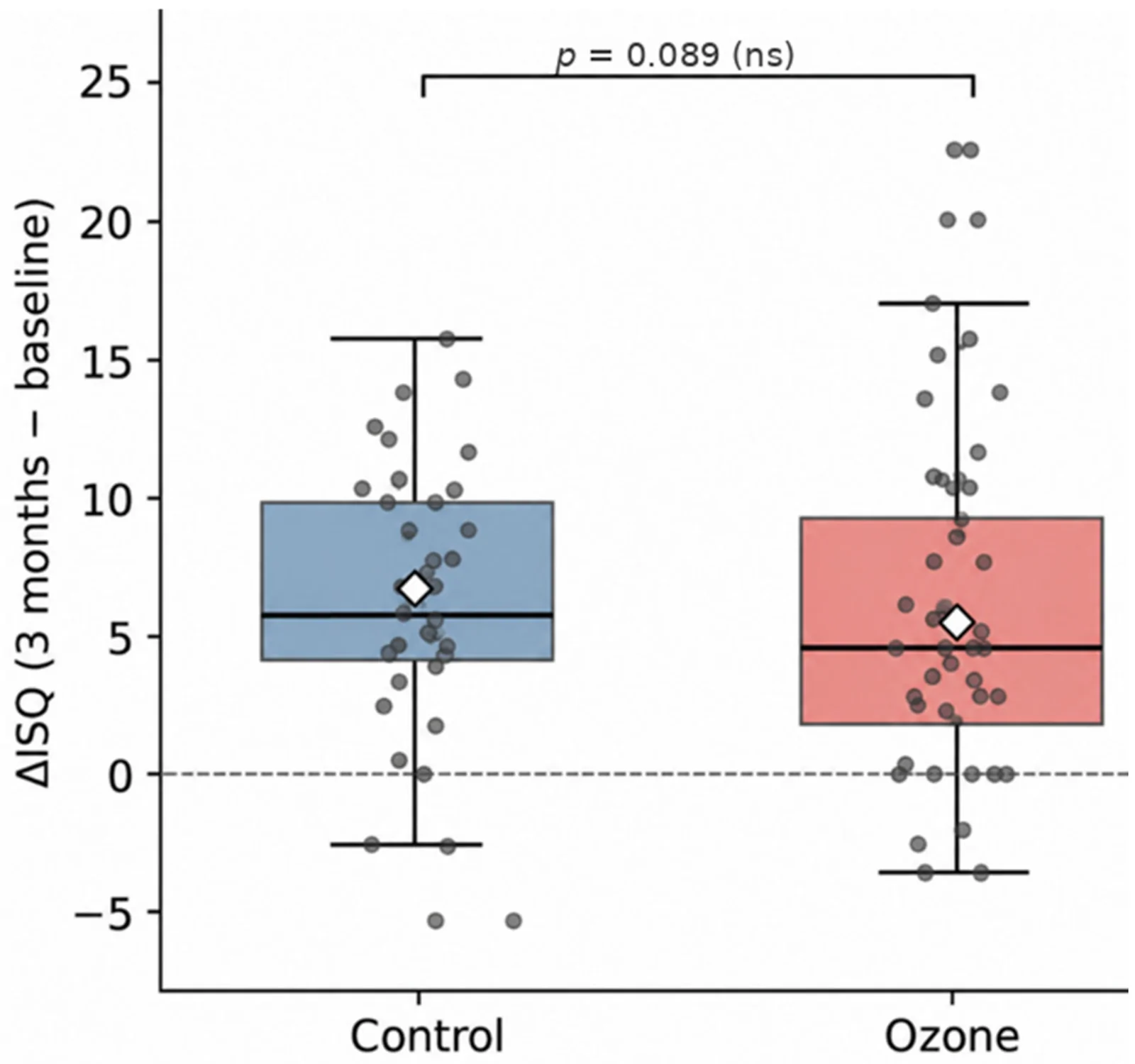
Primary outcome (ΔISQ = 3-month − baseline) by group. Boxplots show the median and interquartile range (IQR), diamonds indicate the mean, and dots represent individual patients. The ozone group showed a greater increase in ISQ than the control group. “ns” indicates a non-significant difference, and the dashed line represents the zero-reference line (ΔISQ = 0).

Implant stability increased significantly from baseline to three months in both groups (Figure 3). However, the increase in ISQ was greater in the ozone group than in the control group. The distributions of ΔISQ according to treatment group are presented in Figure 4.

No significant between-group difference was observed in baseline ISQ (β = −0.49; 95% CI: −2.73 to 1.75; *p* = 0.668). Similarly, insertion torque did not differ significantly between the groups (β = −0.62 Ncm; 95% CI: −1.96 to 0.72; *p* = 0.362). Implant stability increased significantly from baseline to three months in both groups (both *p* < 0.001), with a mean ΔISQ of 5.52 ± 5.82 in the control group and 7.92 ± 5.80 in the ozone group. At three months, ISQ was significantly higher in the ozone group than in the control group (β = 1.91; 95% CI: 0.75 to 3.06; *p* = 0.001). ΔISQ was also significantly greater in the ozone group (β = 2.40; 95% CI: 0.34 to 4.45; *p* = 0.022), corresponding to a small-to-moderate effect size (Cohen’s d = 0.42). In contrast, postoperative pain assessed using the visual analog scale (VAS) did not differ significantly between the groups (β = −0.23; 95% CI: −0.83 to 0.36; *p* = 0.446) (Table 2).

Model assumptions were satisfied for the primary ΔISQ model (Shapiro–Wilk *p* = 0.54; Levene’s *p* = 0.85). In contrast, residuals for three-month ISQ, insertion torque, and VAS showed deviations from normality. Sensitivity analyses showed a consistent direction of effect across the implant-level mixed-effects models, cluster-robust analyses, and patient-level paired analyses. The difference in ΔISQ was statistically significant in the mixed-effects model (*p* = 0.022) and cluster-robust analysis (*p* = 0.036), but not in the patient-level paired analysis (*p* = 0.089). Standardized effect sizes indicated negligible baseline differences between the groups, small-to-moderate effects favoring the ozone group for three-month ISQ and ΔISQ, and no meaningful effect on VAS scores (Table 3 and Figure 4).

**Figure 5:**
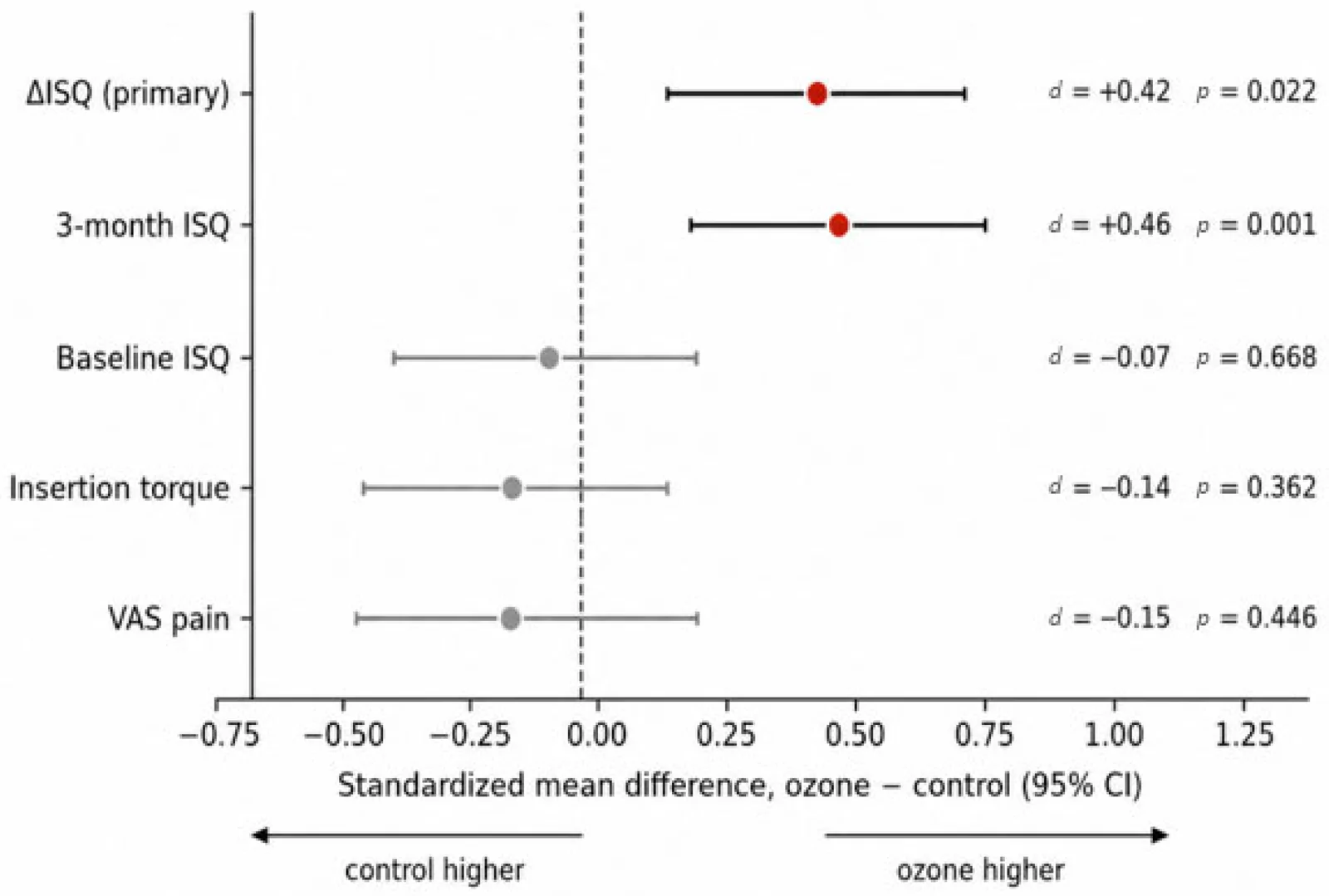
Forest plot of standardized between-group effect sizes (Cohen’s d) derived from the mixed-effects models. Points represent standardized mean differences (ozone − control), and horizontal lines indicate 95% confidence intervals. Positive values indicate higher values in the ozone group. Significant between-group differences were observed for ΔISQ and 3-month ISQ but not for baseline ISQ, insertion torque, or VAS pain. The dashed vertical line represents the null-effect reference (Cohen’s *d* = 0).

Sensitivity analyses showed a consistent direction of effect across the implant-level mixed-effects models, cluster-robust analyses, and patient-level paired analyses. The difference in ΔISQ was statistically significant in the mixed-effects model (*p* = 0.022) and cluster-robust analysis (*p* = 0.036), but not in the conservative patient-level paired analysis (*p* = 0.089). This finding is consistent with the greater statistical efficiency of the mixed-effects model, which uses all implant-level observations, compared with analyses based on patient-level means. Standardized effect sizes indicated negligible baseline differences between the groups, small-to-moderate effects favoring the ozone group for three-month ISQ and ΔISQ, and no meaningful effect on VAS scores (Table 4 and Figure 5).

## 4. Discussion

Maintaining adequate implant stability during the early osseointegration period is an important clinical consideration for successful implant treatment; however, the effectiveness of adjunctive approaches intended to support this process remains uncertain. In the present study, intraoperative ozone application was associated with higher three-month ISQ values and a greater change in ISQ from baseline to three months (ΔISQ), while no significant between-group differences were observed in baseline implant stability, insertion torque, or postoperative pain.

A major gap in the literature is the limited number of controlled clinical studies evaluating the effect of ozone on early osseointegration while controlling for patient- and surgery-related variables, applying ozone directly to the osteotomy, and assessing implant stability using objective methods [15]. Indeed, existing reviews indicate that the literature on ozone in implant dentistry has focused predominantly on surface decontamination in the treatment of peri-implantitis and is characterized by substantial methodological heterogeneity [8,16].

In this context, the split-mouth design enabled each patient to serve as their own control, thereby reducing the influence of potential confounders such as bone quality, systemic status, and individual healing capacity and allowing for a more reliable evaluation of the potential biological effects of ozone [17,18]. Moreover, the analysis of implant-level data using linear mixed-effects models and the validation of the findings using alternative statistical approaches support the robustness of the results [19]. Nevertheless, the modest magnitude of the observed effect and the sensitivity of statistical significance to the analytical approach indicate that the findings should be interpreted as hypothesis-supporting rather than confirmatory. However, these considerations do not exclude the possibility that the observed effect reflects a small but genuine biological contribution.

The fact that the primary outcome reflected a change occurring during the healing period is important when considering the potential biological basis of the observed effect. Ozone has been reported to regulate antioxidant responses through controlled oxidative stimulation, enhance osteoblast activity, promote the expression of osteocalcin and bone morphogenetic proteins, shift the RANKL/OPG balance toward bone formation, and stimulate VEGF-mediated angiogenesis. Its potential effect would therefore be expected to involve bone remodeling during healing rather than primary mechanical stability. Accordingly, the emergence of a difference during healing, rather than at implant placement, is consistent with the proposed biological mechanism [11,12,13]. This interpretation is also supported by preclinical findings. Experimental studies have reported that ozone accelerates early peri-implant bone healing in ovariectomized rats and promotes bone maturation and early angiogenesis in critical-sized bone defects [20,21]. However, these studies relied predominantly on histomorphometric and immunohistochemical outcomes and demonstrated their effects mainly in osteoporotic or critical-sized defect models. Therefore, effects of a similar magnitude may not be expected for clinical implant stability in healthy bone with high initial stability. Clinical evidence evaluating early implant stability as the primary outcome of ozone application remains limited [8], suggesting that the apparent agreement between preclinical and clinical findings may be based on different biological contexts. Potential sources of variation in effect magnitude across studies include the form, dose, and duration of ozone exposure, as well as bone quality and follow-up duration [22].

Within this context, the clinical relevance of the primary finding should be interpreted cautiously. When considered in relation to the measurement variability inherent in resonance frequency analysis and the thresholds used to guide loading decisions, a difference of approximately two ISQ units, even if genuine, is unlikely to be clinically decisive [23]. The higher absolute implant stability observed in the ozone group at three months suggests that the effect of ozone may emerge during the healing process. However, this finding should be interpreted cautiously. At high stability levels, the ISQ scale may exhibit a ceiling effect, and the high ISQ values reached by both groups may complicate the assessment of the clinical relevance of the between-group difference [5,6]. Moreover, absolute ISQ differences of this magnitude may fall within the measurement variability of the device. Therefore, it remains uncertain whether the higher ISQ values observed at three months reflect stronger osseointegration or merely earlier attainment of a stability plateau. This finding should consequently be regarded as a supportive secondary observation rather than independent evidence of clinical superiority.

The absence of significant between-group differences in indicators of primary stability at implant placement and in early postoperative pain supports the interpretation that the observed effect may be related to biological processes during healing rather than surgical or mechanical factors. This interpretation is consistent with the prevailing view that primary implant stability is determined mainly by mechanical factors, whereas secondary stability is associated with biological healing. No significant between-group difference was observed in postoperative pain [24]. A single ozone application confined to the implant osteotomy may have been insufficient to produce the analgesic effects reported with repeated soft-tissue applications [11]. Furthermore, because both surgical sites were present within the same patient under the split-mouth design, the diffuse intraoral perception of pain and patients’ difficulty in distinguishing between the two sides may have reduced the ability to detect small between-group differences.

The findings should be considered in the context of the existing literature on adjunctive approaches aimed at enhancing implant stability and osseointegration. In addition to modifications of implant macrogeometry, surface treatments designed to alter surface roughness, chemistry, and hydrophilicity have been widely investigated to improve bone–implant interactions and promote early bone formation [25]. Physical adjuncts, including photobiomodulation and low-level laser therapy, have also been evaluated for their potential effects on peri-implant bone healing and secondary stability, although clinical evidence remains heterogeneous [26,27]. Current evidence further indicates that no approach among mechanical, chemical, and physical decontamination methods has demonstrated clear clinical superiority, and no universally accepted gold standard has been established [7,8]. A potential advantage of ozone over these approaches is its capacity to biologically support healing in addition to its antimicrobial activity [11]. However, the present study cannot determine whether the modest improvement in stability resulted from microbial decontamination or direct biological effects.

From a clinical perspective, a single intraoperative ozone application appears to be a straightforward and well-tolerated intervention. Nevertheless, the modest magnitude of the observed effect is insufficient to support its routine clinical use or changes to existing loading protocols. Because the findings suggest that the potential contribution of ozone may be related more closely to healing biology than to primary mechanical stability, future studies should investigate whether its clinical benefit is more pronounced in populations at increased risk of impaired osseointegration, including patients with diabetes, smokers, individuals with poor bone quality, and implants placed in augmented sites [20,21].

These findings should be interpreted in light of several limitations. The three-month follow-up allowed for assessment of only the early healing period and precluded conclusions regarding implant survival, marginal bone loss, and long-term clinical success. Furthermore, the statistical advantage of the split-mouth design may have been limited by the low within-patient correlation observed for the primary outcome, potentially contributing to differences among the analytical approaches [28]. The single-center setting and use of a single implant system and ozone application protocol limit the generalizability of the findings. Finally, because the proposed biological effects of ozone were not confirmed using histological or microbiological data, the underlying mechanisms remain inferential [29]. Therefore, larger multicenter studies evaluating long-term clinical outcomes, different ozone application protocols, and potential biological mechanisms are needed.

## 5. Conclusions

In this randomized controlled split-mouth study, intraoperative ozone gas application to the implant osteotomy immediately before implant placement was associated with a greater increase in the implant stability quotient (ΔISQ) than the control protocol. The higher ISQ values observed in the ozone group at three months further supported this finding. In contrast, postoperative pain did not differ significantly between the groups.

These findings suggest that intraoperative ozone application may contribute to the development of secondary implant stability by supporting biological processes during early osseointegration. However, given the small magnitude of the observed effect and the sensitivity of statistical significance to the analytical approach, the current findings are insufficient to support changes to routine clinical treatment protocols.

To our knowledge, this is among the first clinical studies to evaluate the effect of intraoperative ozone gas application to the implant osteotomy on early implant stability using a randomized split-mouth design and resonance frequency analysis. The findings contribute to the existing evidence regarding the potential role of ozone in implant osseointegration. Nevertheless, larger multicenter randomized controlled trials with extended follow-up are required to confirm the clinical relevance of this effect and determine its applicability across different patient populations.

## Figures and Tables

**Table 1:** Participant Demographics and Implant Characteristics.

Characteristic	Value
Patient-level (*n* = 40)	
Age, years—mean ± SD (range)	46.0 ± 11.1 (24–65)
Sex—*n* (%)	
Female	20 (50.0)
Male	20 (50.0)
Implant-level (*n* = 106)	
Implants per patient—mean ± SD (range)	2.65 ± 1.46 (2–8)
Group allocation—Ozone/Control, *n*	53/53
Jaw—*n* (%)	
Maxilla	64 (60.4)
Mandible	42 (39.6)
Implant diameter—4.0/4.5 mm, *n*	101/5
Implant diameter 4.0 mm—*n* (Ozone/Control)	101 (51/50)
Implant diameter 4.5 mm—*n* (Ozone/Control)	5 (2/3)
Implant length—10/12 mm, *n*	99/7
Implant length 10 mm—*n* (Ozone/Control)	99 (49/50)
Implant length 12 mm—*n* (Ozone/Control)	7(4/3)
Number of implants contributed per patient—*n* patients (Ozone/Control)	106 (53/53)
1 implant	32/32
2 implants	5/5
3 implants	1/1
4 implants	2/2

Data are presented at the patient or implant level, as appropriate. In the split-mouth design, the same 40 patients contributed implants to both treatment groups. SD, standard deviation.

**Table 2:** Primary analysis: linear mixed-effects models (implant-level analysis, *n* = 106 implants; random intercept for patient).

Outcome	Control (Mean ± SD)	Ozone (Mean ± SD)	β (Ozone − Control) (95% CI)	ICC	*p*
Baseline ISQ (mean)	69.84 ± 6.58	69.35 ± 7.99	−0.49 (−2.73 to 1.75)	0.32	0.668
Insertion torque (Ncm)	32.55 ± 4.17	31.92 ± 4.59	−0.62 (−1.96 to 0.72)	0.34	0.362
3-month ISQ (mean)	75.36 ± 4.41	77.26 ± 4.04	+1.91 (0.75 to 3.06)	0.46	0.001
ΔISQ—primary	5.52 ± 5.82	7.92 ± 5.80	+2.40 (0.34 to 4.45)	0.12	0.022
VAS pain (0–10)	5.02 ± 1.18	4.79 ± 1.91	−0.23 (−0.83 to 0.36)	0.04	0.446

Values are presented as mean ± SD at the implant level (Ozone, *n* = 53; Control, *n* = 53). β represents the fixed-effect estimate (ozone − control) from the linear mixed-effects model, evaluated using the Wald test. ICC, intraclass correlation coefficient; ISQ, implant stability quotient; SD, standard deviation; VAS, visual analog scale. Standardized effect sizes (Cohen’s d; ozone − control) were baseline ISQ, d = −0.07; insertion torque, d = −0.14; 3-month ISQ, d = +0.46; ΔISQ, d = +0.42; and VAS pain, d = −0.15.

**Table 3:** Sensitivity Analysis Across Three Analytical Approaches (ozone − control).

Outcome	β (Ozone − Control)	Patient-Level Paired, *p*	Mixed Model, *p*	Cluster-Robust, *p*
Baseline ISQ	−0.49	0.892	0.668	0.731
Insertion torque	−0.62	0.682	0.362	0.440
3-month ISQ	+1.91	<0.001	0.001	0.007
ΔISQ—primary	+2.40	0.089	0.022	0.036
VAS pain	−0.23	0.903	0.446	0.502

All three analytical approaches yielded estimates in the same direction. For the primary outcome (ΔISQ), the implant-level mixed-effects model and cluster-robust analysis yielded *p* = 0.022 and *p* = 0.036, respectively, whereas the patient-level paired analysis yielded *p* = 0.089. The patient-level analysis was based on the mean implant-level value for each treatment side within each patient. The patient-level comparison of 3-month ISQ was performed using the Wilcoxon signed-rank test.

**Table 4:** Standardized Effect Sizes and Sensitivity Analysis.

Between-Group Outcome (Ozone − Control)	Standardized Effect Size (Cohen’s d)	Interpretation
Baseline ISQ	−0.07	Negligible; groups equivalent at baseline
Insertion torque	−0.14	Negligible; groups equivalent at baseline
3-month ISQ	+0.46	Small-to-moderate effect favoring ozone
ΔISQ—primary	+0.42	Small-to-moderate effect favoring ozone; significant in the LMM and cluster-robust analyses, but not in the patient-level paired analysis
VAS pain	−0.15	Negligible; credible absence of difference

A sensitivity analysis of the patient-level paired comparison showed that 40 participants provided 80% power to detect a minimum standardized paired effect size of *Cohen’s dz* = 0.45 at a two-sided significance level of 0.05. The implant-level linear mixed-effects model incorporated all 106 implant observations while accounting for clustering within participants. The standardized implant-level effect for ΔISQ was *Cohen’s d* = 0.42. Statistical significance differed across analytical approaches: the effect was significant in the linear mixed-effects and cluster-robust analyses, but not in the patient-level paired analysis.

## Data Availability

The datasets generated and/or analyzed during the current study are available from the corresponding author upon reasonable request.

## Abbreviations

BMP	Bone Morphogenetic Protein
CI	Confidence Interval
ICC	Intraclass Correlation Coefficient
ISQ	Implant Stability Quotient
LMM	Linear Mixed-Effects Model
N·cm	Newton-centimeter
NCT	ClinicalTrials.gov Identifier
OLS	Ordinary Least Squares
OPG	Osteoprotegerin
RFA	Resonance Frequency Analysis
RANKL	Receptor Activator of Nuclear Factor Kappa-B Ligand
REML	Restricted Maximum Likelihood
SD	Standard Deviation
VAS	Visual Analog Scale
VEGF	Vascular Endothelial Growth Factor
β	Regression Coefficient
ΔISQ	Change in Implant Stability Quotient (3-month ISQ − baseline ISQ)
